# Identification of genetic markers with synergistic survival effect in cancer

**DOI:** 10.1186/1752-0509-7-S1-S2

**Published:** 2013-08-12

**Authors:** Riku Louhimo, Marko Laakso, Tuomas Heikkinen, Susanna Laitinen, Pekka Manninen, Vladimir Rogojin, Minna Miettinen, Carl Blomqvist, Jianjun Liu, Heli Nevanlinna, Sampsa Hautaniemi

**Affiliations:** 1 Systems Biology Laboratory, Genome-Scale Biology Research Program, University of Helsinki, Helsinki, Finland; 2 CSC - IT Center for Science Ltd, Espoo, Finland; 3 Department of Obstetrics and Gynecology, Helsinki University Central Hospital, Helsinki, Finland; 4 Human Genetics, Genome Institute of Singapore, Singapore, 60 Biopolis Street 02-01 Singapore 138672; 5 Department of Oncology, Helsinki University Central Hospital, Helsinki, Finland

## Abstract

**Background:**

Cancers are complex diseases arising from accumulated genetic mutations that disrupt intracellular signaling networks. While several predisposing genetic mutations have been found, these individual mutations account only for a small fraction of cancer incidence and mortality. With large-scale measurement technologies, such as single nucleotide polymorphism (SNP) microarrays, it is now possible to identify combinatorial effects that have significant impact on cancer patient survival.

**Results:**

The identification of synergetic functioning SNPs on genome-scale is a computationally daunting task and requires advanced algorithms. We introduce a novel algorithm, Geninter, to identify SNPs that have synergetic effect on survival of cancer patients. Using a large breast cancer cohort we generate a simulator that allows assessing reliability and accuracy of Geninter and logrank test, which is a standard statistical method to integrate genetic and survival data.

**Conclusions:**

Our results show that Geninter outperforms the logrank test and is able to identify SNP-pairs with synergetic impact on survival.

## Introduction

Cancer is a complex disease that develops from accumulated genetic mutations that impair cellular processes responsible for maintaining homeostasis. For instance, inherited breast cancer predisposition is currently thought to result from rare high penetrance mutations in high risk families, or multiplicative effects of moderate penetrance variants or common low risk variants in the population [1,2]. So far, over 20 low penetrance variants, such as single nucleotide polymorphisms (SNPs), have been identified but they only explain approximately 8% of the familial risk of breast cancer, with the high and moderate penetrance genes explaining roughly 25% [3,4]. Combinatorial effects of large numbers of putative risk alleles are likely to be important in further explaining the genetic risk for breast cancer [5]. Increasing evidence suggests that not only breast cancer risk but also prognosis is inherited, and germline variants have been found to associate with survival of cancer patients [6]. Furthermore, interactive survival effects of genetic variants from cancer pathways have also been implicated [7], and survival effects detected for specific genotype carriers after defined chemotherapy treatment indicate treatment resistance conferred by inherited genetic variation [8]. However, few studies up to now have analyzed genome-wide the combinatorial survival effects of polymorphisms interacting with each other or with clinical features [7,9,10]. The large-scale analysis of interactive effects between genetic markers, or between genetic markers and clinical variables, will be important in increasing our understanding of diseases like cancer [11]. Uncovering these combinatorial survival effects will provide new markers for clinical decision making and personalized treatment of cancer patients [5].

Identification of markers that have combinatorial survival effect requires an iterative systems biology approach with efficient computational methodology which can be executed on high-performance computing clusters [12]. Here we introduce a novel algorithm, Geninter, for discovering interacting SNPs with combinatorial survival effect, *i.e*., SNPs that individually have no survival effect but together contribute significantly to survival. Previous efforts in discovering specific combinatorial genotypes have focused on small, highly selected groups of SNPs [9,10], and to our knowledge Geninter is the first algorithm that is able to systematically integrate SNP-pairs with survival data on a genome-wide scale.

## Methods

**Figure 1:**
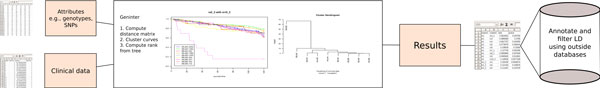
**The outline of the Geninter analysis workflow**. First, an attribute matrix containing genotypes and a matrix of survival times are given as an input to Geninter. The analysis is divided into three steps. The results (sorted list of SNP-pairs) can then be 12 annotated, for instance, using the Ensembl database and filtered to exclude markers that are in linkage disequilibrium (LD). The output contains the marker pairs, their ranks and p-values.

Genome-wide analysis of pair-wise SNPs brings forward two major challenges. First, the combination of multiple marker genotypes increases the number of groups in the survival analysis. The major consequences of the increased number of groups are that (i) the number of samples should be relatively high in order to ensure stable estimates in the subgroups, and (ii) the increase in the number of survival curves leads to more intersections of the curves, which renders the logrank statistic less reliable [13]. This issue is exacerbated by the tendency of the logrank test to overestimate large cohorts to have significant survival differences even when the difference is only slight. Second, SNP microarrays produce states for hundreds of thousands or millions of markers making evaluation of all the pairs computationally intensive [11]. Geninter addresses the computational challenges with optimized code and distributed programming. The overall outline of Geninter is given in Figure 1. Here we provide details on how each step in Geninter is executed. First, an attribute matrix containing genotypes and a matrix of survival times are given as an input to Geninter. The analysis is divided into three steps: (1) determining the distance matrix based on the genotype combination specific Kaplan-Meier curves; (2) using hierarchical clustering to determine the underlying relative structure of the curves; and (3) computing the rank. If the rank of a SNP-pair exceeds a chosen threshold, the pair is considered as a putative survival affecting combination and stored. The user can define the threshold parameter based on the number of SNP-pairs or p-value cutoff. We have implemented Geninter so that it can be run as an individual program but also on Anduril bioinformatics workflow engine that allows advanced processing of the Geninter results, such as automated annotation (*e.g.*, linkage disequilibrium (LD) mapping) from bio-databases [14].

### Determining the distance matrix

The first stage of Geninter is the calculation of a distance matrix D  for a family of Kaplan-Meier survival curves. A family of curves is the set of curves for which one instance of the statistic is calculated. The Kaplan-Meier estimate for surviving to at least time *t_j _*is equal to the conditional probability of surviving beyond *t_j _*multiplied by the estimate at the previous time point *t_j−1_*. At time 0, all patients are alive. The area between curves was chosen as the distance metric because it is (1) robust to possible erratic behavior of curve functions, and (2) computationally simple. Let *C *= {*c*_1_, *c*_2_, ..., *c_m_*} be a set of *m *survival curves. For example, for a SNP-pair, *m *∈ [1,9] since there are three alleles for each SNP (*e.g.*, AA, AB and BB) and thus 9 possible combinations of alleles. Let *c_j _*and *c_k _*be survival curves and *c_j_*, *c_k _*∈ *C*. For every time point *t_i_*, where *i *∈ [2, *n*] and *n *is the total number of time points available in follow-up, we calculate the distance between the survival curves as

(1) $$D\left(c_{j},c_{k}\right)=\overset{n}{\underset{i=2}{\sum }}\left(t_{i}-t_{i-1}\right)|S_{c_{j}}\left(i\right)-S_{c_{k}}\left(i\right)|,$$

where $S_{c_{j}}\left(i\right)=c_{j}\left(t_{i-1}\right)$ and $S_{c_{k}}\left(i\right)=c_{k}\left(t_{i-1}\right)$ denote the survival rates of the curves *c_j _*and *c_k _*at the given time point. To determine the distance matrix for a family of survival curves, all pairwise distances *D *(*c_j_*, *c_k_*) are calculated to form the distance matrix D . *D *can be thought to correspond to the sum of areas of rectangles

$$\left(t_{i},S_{c_{j}}\left(i\right)\right),\left(t_{i},S_{c_{j}}\left(i\right)\right),\left(t_{i-1},S_{c_{k}}\left(i\right)\right),\left(t_{i-1},S_{c_{k}}\left(i\right)\right),\;\;\forall i\in \left[2,n\right].$$

### Hierarchical clustering

**Figure 2:**
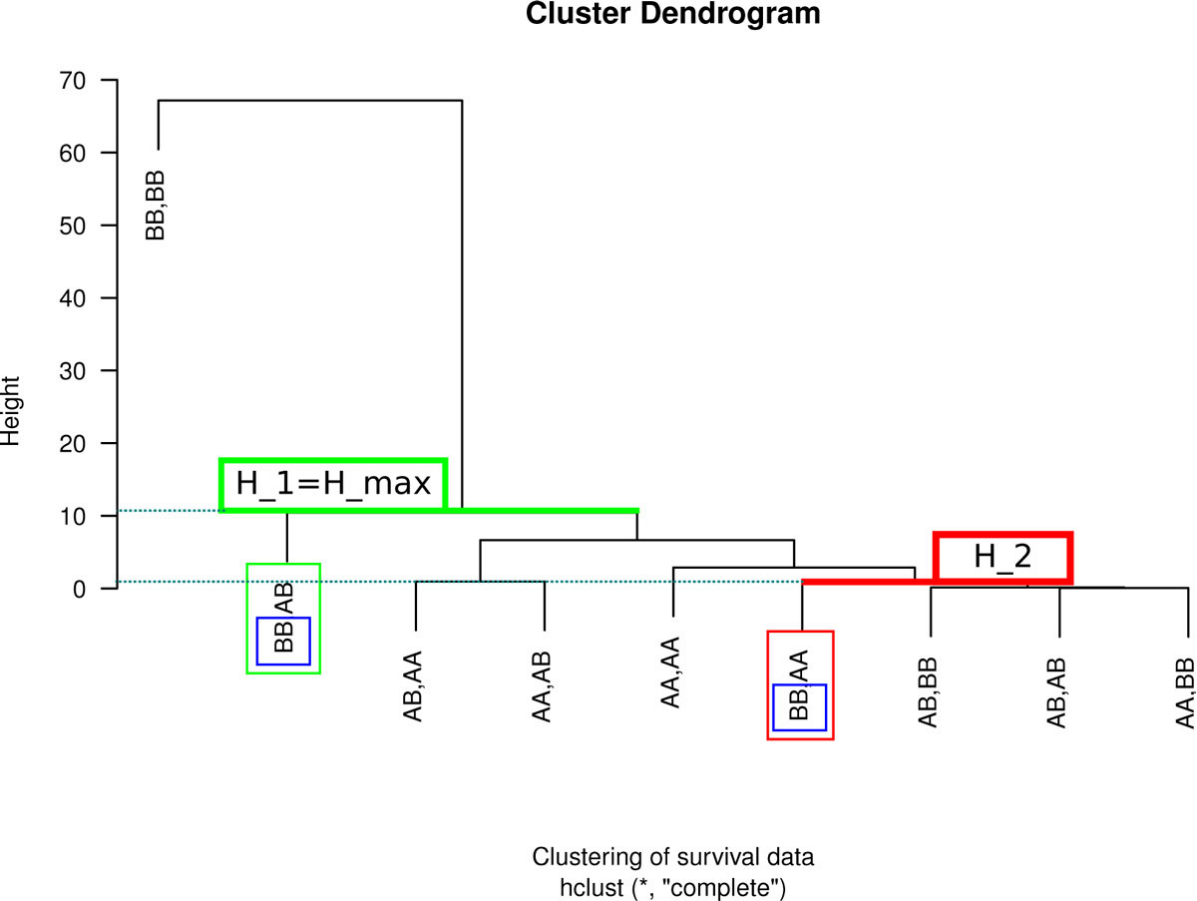
**Cluster tree dendrogram for a family of curves and the distance of two alleles**. The features (alleles) circled in blue satisfy the equivalence relation. The allele combinations circled in green and red have a distance of |*H_max _*− *H*_2_|. The height of each leaf node is the height of its parent. This is marked with the blue dashed lines for the circled leafs.

In the second stage, curves in a family are clustered by complete linkage agglomerative hierarchical clustering using D  as the distance matrix. The main benefit of the hierarchical clustering is a dendrogram in which leafs are clusters, the leafs contain biological information which can be taken advantage of, and the clusters represent survival curves (Figure 2). In the complete linkage the distances between two clusters are calculated as the maximum distance between any object in the first and any object in the second cluster. We chose complete linkage clustering over single or average linkage because it more effectively distinguishes curves that are farthest away from one another. However, Geninter allows its user to define any alternative method supported by the underlying clustering library.

### Cluster tree distance

Curves in a curve family correspond to unique combinations of features (*e.g.*, alleles). Each combination of features contains *e *features constrained by *e *domains respectively (*e.g.*, SNP markers). Formally, a curve *c_j _*corresponds to a tuple of features (*a_j,1_*, *a_j,2_*, . . . , *a_j,e_*) over the cartesian product of the feature domains *A*_1 _× *A*_2 _× . . . × *A_e_*. For the set of curves *C *= {*c*_1_, *c*_2_, . . . , *c_m_*} and its corresponding feature combinations we define its attributes *M_j_*, 1 ≤ *j *≤ *e *as vectors such that *M_j _*= (*a*_1,*j*_, *a*_2,*j*_, . . . , *a_m,j_*). In other words, for each domain *A_j _*we define its attribute *M_j _*which represents features from the domain *A_j _*corresponding to all the curves from *C*.

For example, let us have two SNP markers such that SNP1 has one allele BB and SNP2 has two alleles AA and AB. Thus, we have two domains *A*_1 _= {*BB*} and *A*_2 _= {*AA*, *AB*}. We can have the following SNP-pair combinations

(2) $$\begin{array}{l} \underline{\begin{array}{cc} \;\;\;\text{SNP}1(M_{1}) & \text{SNP}2(M_{2}) \end{array}}\\ \begin{array}{cc} \begin{array}{cc} c_{1} & \;BB\\ c_{2} & \;BB \end{array}\;\;\;\; & \begin{array}{c} AA\\ AB \end{array} \end{array} \end{array}$$

In this way, for SNP1 we have attribute *M*_1 _= (*BB*, *BB*) and for SNP2 we have attribute *M*_2 _= (*AA*, *AB*).

In general, we can represent combinations of *e *features corresponding to *m *curves as a matrix:

$$M=\left(\begin{array}{cccc} a_{1,1} & a_{1,2} & \cdots \; & a_{1,e}\\ a_{2,1} & a_{2,2} & \cdots \; & a_{2,e}\\ \vdots & \vdots & \ddots & \vdots \\ a_{m,1} & a_{m,2} & \cdots \; & a_{m,e} \end{array}\right)=\left(M_{1}^{T}\;M_{2}^{T}\;\;\;\;\cdots \;\;M_{e}^{T}\right),$$

**Figure 3:**
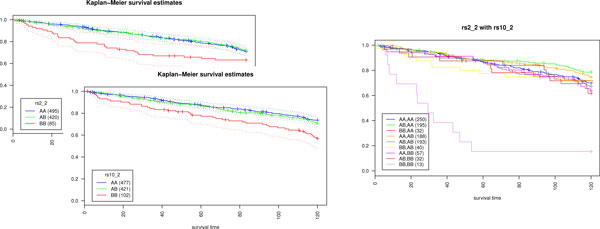
**Combinatorial genotypic survival effect**. Combination of two markers reveals synergetic effect on survival. The panel inside each figure contains the genotypes and sample sizes. In the right hand survival image, the curve furthest apart (BB, BB) from the rest is for the first marker most distant from curve (BB, AA) and for the second marker from curve (AB, BB).

For each attribute *M_k_*, 1 ≤ *k *≤ *e *we establish the equivalence relation between curves $c_{j_{1}}$, $c_{j_{2}}\in C$ as follows: $c_{j_{1}}\equiv_{M_{k}}c_{j_{2}}$ if and only if $a_{j_{1}}k=a_{j_{2}}k$. For example, the two allele combinations (*BB*, *AA*) and (*BB*, *AB*) share the feature *BB *in their attribute *M*_1 _for which we can define the equivalence relation $c_{1}\equiv_{M_{1}}c_{2}$ (see Figures 3 and 2). Let $E_{M_{j}}$ be a set of equivalence classes for $\equiv_{M_{j}}$ and let $E_{M_{j}}$ have *l_j _*equivalence classes $\left\{E_{1,j},E_{2,j},\cdots ,E_{\text{l},j}\right\}$ (note that *l_j _*≤ *m*). We can define the distance within an equivalence class with the cluster dendrogram.

Let *H*_1 _be the height in the dendrogram tree of the cluster nearest to $c_{j_{1}}$ and *H*_2 _similarly for $c_{j_{2}}$. *H_max _*is defined as the height in the dendrogram of the smallest cluster (last common ancestor) into which both $c_{j_{1}}$ and $c_{j_{2}}$ belong. Then, provided that $c_{j_{1}},c_{j_{2}}\in C$ and $c_{j_{1}}\equiv_{M_{j}}c_{j_{2}}$, the distance between two curves in the cluster tree is

(3) $$d\left(c_{j_{1}},c_{j_{2}}\right)=\left\{\begin{array}{cc} \left|H_{max}-H_{1}-H_{2}\right| & \text{if }H_{max}\neq H_{1},H_{max}\neq H_{2}\\ \left|H_{max}-H_{1}\right| & \text{if }H_{max}=H_{2}\\ \left|H_{max}-H_{2}\right| & \text{if }H_{max}=H_{1}\\ 0 & \text{if }H_{max}=H_{1}=H_{2} \end{array}\right)$$

One possible distance *d *that satisfies the equivalence relation is shown in Figure 2. The distance *d *in the family of survival curves does not exceed the maximum survival rate 1.0 multiplied by the last time point, *i.e*., 0 ≤ *d *≤ *t_n_*.

Now, we can define the maximal distance between two curves in the equivalence class $E_{k,j}$

(4) $$d_{E_{k,j}}=\underset{c_{j1},c_{j2}\in \;E_{k,j}}{\text{max}}\left(d\left(c_{j1},c_{j2}\right)\right).$$

### Rank calculation

Every curve depicts the survival of a group sharing a combination of attributes. For example, the SNP-combinations (BB, AA) and (BB, AB) contain the attributes (*BB*, *BB*) and (*BB*, *AB*) as depicted in Matrix 2. The rank of a single attribute is the maximum of its cluster tree distances that satisfy the equivalence relation. In the final step, we calculate one rank for each attribute, sum the attribute ranks, and compute the final rank as the average of these partial ranks.

The partial rank corresponding to an attribute (*i.e.*, the rank of a single marker or other attribute) over all curves is defined by the maximum distance of all the different equivalence classes

(5) $$R_{M_{j}}=\underset{E_{k,j}\in E_{M_{j}}}{\text{max}}\left(d_{E_{k,j}}\right).$$

Given the last time point *t_n_*, the rank of the family of survival curves is the sum of all the partial ranks

(6) $$\bar{R}=\frac{\sum_{j=1}^{e}R_{M_{j}}}{et_{n}}.$$

For example, the rank of two markers SNP1 and SNP2 is

$$\bar{R}=\frac{\left(R_{SNP1}+R_{SNP2}\right)}{2t_{n}}.$$

This formulation of the rank allows us to extend the algorithm to multiple combinations of attributes. Moreover, the attributes are not constrained to be SNP-markers or clinical variables but can be anything for which we can define an equivalence relation.

### Rank distribution

In order to study the properties of the Geninter rank distribution, we generated a data set with 1,000 patients, 140 markers, and uniformly distributed survival times. Under the null hypothesis of "no survival effect", we observed that the density function of the rank distribution could be approximated by a gamma distribution. We tested altogether 18 statistical distributions including Gaussian, log-normal and binomial. The best fitting distribution using log-likelihood was the three-parameters generalized extreme value distribution followed by the two-parameter gamma distribution. As the gamma distribution consisted of only two parameters, we chose that to represent the data. The gamma distribution approximation enables us to compute p-values in constant time for every rank statistic.

Assume that the rank $\bar{R}$ is a Gamma distributed random variable. Then $\bar{R}\sim \text{Γ}\left(k,\theta \right)$, where *k *and *θ *are the shape and scale, respectively. Let $\hat{\mu }$ be the sample mean of the distribution and ${\hat{\sigma }}^{2}$ the sample variance. Since the maximum likelihood estimator for the scale parameter is $\hat{\theta }=\frac{\hat{\mu }}{k}$, and it is known that ${\hat{\sigma }}^{2}=k{\hat{\theta }}^{2}=k{\left(\frac{\hat{\mu }}{k}\right)}^{2}=\frac{{\hat{\mu }}^{2}}{k}$, it follows that

$$k=\frac{{\hat{\mu }}^{2}}{{\hat{\sigma }}^{2}},\;\;\hat{\theta }=\frac{\hat{\mu }}{{\hat{\sigma }}^{2}}.$$

The rank distribution is sensitive to the population size. Therefore, we suggest that the scale and shape parameters are calibrated in respect to the population size. The calibration can be achieved by recalculating $\hat{\theta }$ and *k *for the new null rank distribution.

### Implementation

We have implemented Geninter in the R statistical language, and in Fortran using the Message Passing Interface (MPI) for parallelization [15]. The Fortran/MPI implementation was developed and tested with a HP CP4000 BL ProLiant cluster system of CSC - IT Center for Science Ltd. utilizing the IMSL Fortran Math library for survival computations. Both implementations of the algorithm are freely available at the project website http://csbi.ltdk.helsinki.fi/pub/geninter.

### Breast cancer data description

Genotype data were obtained on Finnish breast cancer patients genotyped as described previously [16]. Briefly, the patient set comprised two series of unselected breast cancer patients and additional familial cases diagnosed at the Helsinki University Central Hospital (HUCH).The first patient set was collected in 1997-1998 and 2000 and covers 79% of all consecutive, newly diagnosed cases during the collection periods [17,18]. The second set, containing newly diagnosed patients, was gathered in 2001-2004 and covers 87% of all breast cancer patients treated at HUCH during the collection period [8]. Additional familial cases were collected as described in [19].

## Results

### Cancer genotype-survival simulator

To assess whether Geninter is able to detect true and false positive SNP-pairs we generated a simulator based on 1,000 breast cancer samples chosen from a cohort of breast cancer patients and controls genotyped on the HumanHap550 SNP microarrays [16]. We estimated the genotype frequencies using these 1,000 samples and randomly chosen 150 SNPs. We limited our analysis to those markers where the minor allele frequency exceeded 5%. This resulted in 139 qualifying markers whose genotype frequencies exceeded the threshold. The qualifying genotype frequencies were used as probabilities when generating the simulated markers.

We assumed that under the null hypothesis the survival times are uniformly distributed with the maximum survival of 360 months and the mean survival of 180 months. This assumption gives results in survival curve families with a ten year survival of approximately 75%, which is similar to the ten year overall survival of patients diagnosed with breast cancer in the Nordic countries [20].

In order to introduce predetermined survival effects into the survival times, we randomly chose two markers. Then, samples with the combination of rare homozygotes from both markers were assigned survival times from a logarithmic distribution with a mean of *log *$\left(\frac{360}{16}\right)$. This was then repeated for a different pair of markers in order to create affected marker pairs as defined by the user. A marker could only appear in one affected marker pair in the data generation process. In order to simulate censoring, which is present in all cancer cohorts, we generated random censoring events by choosing events to occur in 80% and censoring in 20% of the samples.

### Analysis and comparison of the simulated data

**Figure 4:**
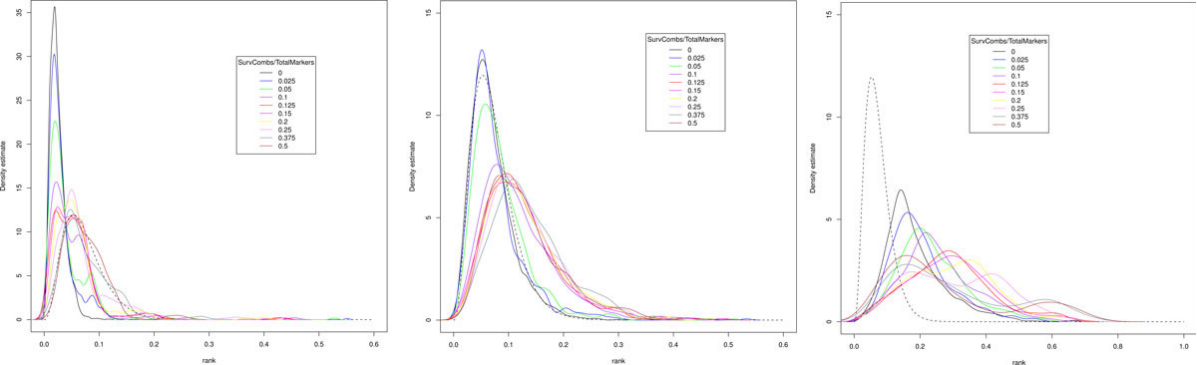
**Effect of population size on rank distribution**. 780 marker combinations have been evaluated for each distribution. The black dashed curve is the hypothetical null distribution. The boxes on the right of each set of curves indicate the ratio of affected and non-affected markers for each curve. If the ratio is 0.5, every marker combination has some induced survival effect. In the left panel population size is 10,000, in the middle panel 1,000, and in the right panel 100.

Logrank test is a well-established statistical method to associate a SNP to survival. We tested both Geninter and logrank test with the simulated data in which the ground truth is known. Based on simulations on the effect of of population size on rank distribution (Figure 4), we estimated the background rank distribution from a simulated cohort of 1,000 samples and used the estimated distribution to compute p-values for the ranks. We applied the false discovery rate (FDR) procedure for the multiple hypothesis correction of the p-values [21]. We verified that the simulated distribution is similar to one calculated from a larger run with real data (data not shown). We further varied the size of our marker set between 40 and 140 markers. The number of marker combinations in the simulation was restricted to 140 because the analysis of 10,000 combinations $\left(\left(\begin{array}{c} 140\\ 2 \end{array}\right)=9730\right)$ does not yet require a high-performance cluster. Our simulator allows controlling the true positives, *i.e*., the marker pairs whose survival times were drawn from the logarithmic distribution.

In order to study the effect of the number of affected markers on the rank statistic, we varied the fraction of affected markers from 1% to 50% of the total marker population. Figure 4 shows how increasing the ratio of affected markers to non-affected markers shifts the rank distribution to the right. With low numbers of affected markers the rank distribution is nearly identical to the background distribution. The 50% fraction represents a pathological case where half of the marker population has some induced survival effect and therefore every marker pair has at least one marker with a survival effect.

**Figure 5:**
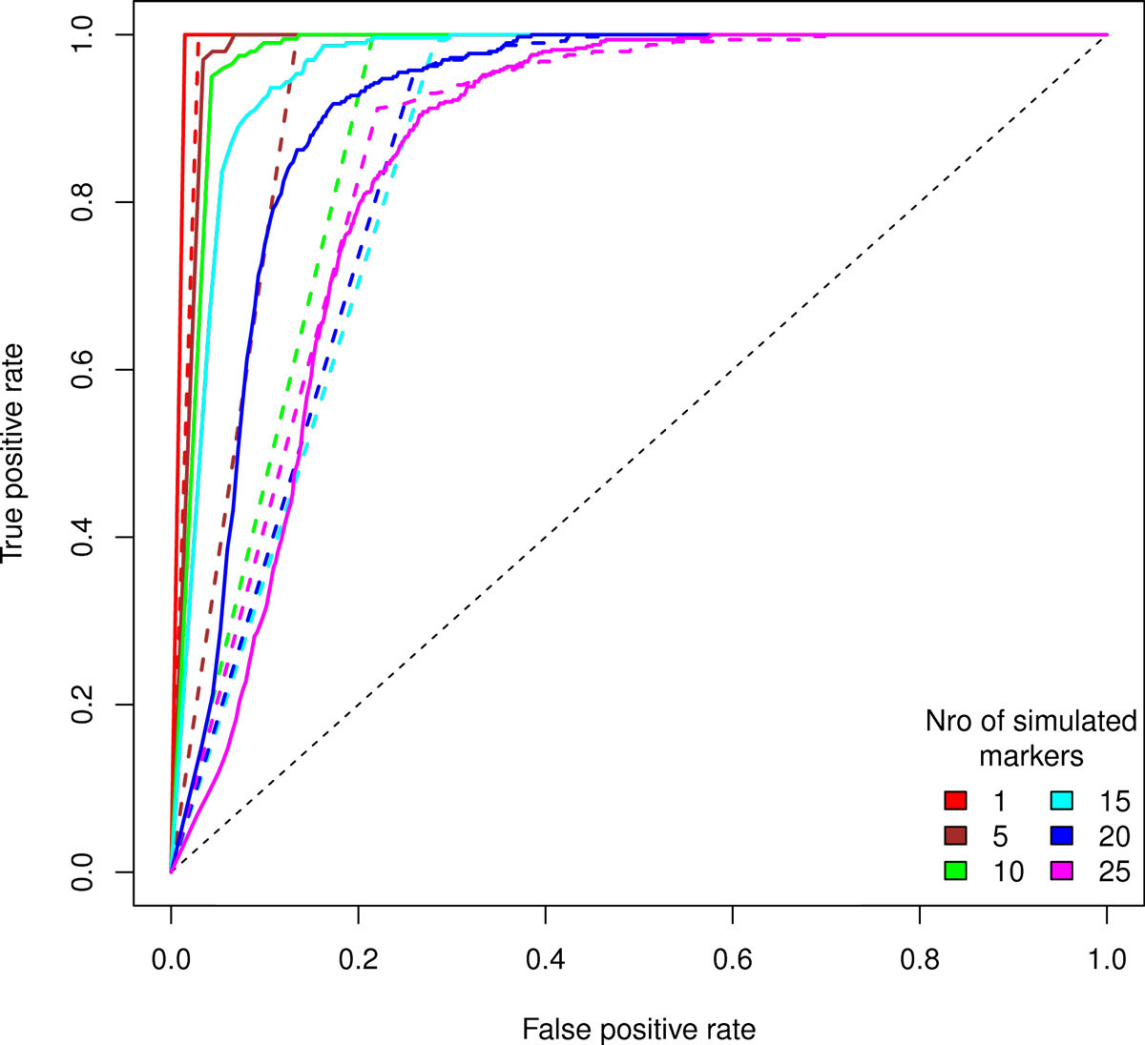
**Average ROC curves for different statistics when the number of affected pairs increases**. Solid lines are computed from the Geninter rank p-values, dashed from the logrank test p-values. Results from simulations with different numbers of affected marker pairs are drawn with different colors. Each curve is an average (averaged over 20 repetitions) of an analysis of a cohort of 10,000 samples with the number of affected marker combinations varying between 1 and 20.

We applied the Geninter and logrank methods to analyze all the combinatorial SNP-SNP survival effects in simulated data. Additionally, we calculated the single SNP survival effects with the logrank test. Evinced in Figure 3, a combination acquires the rank of > 0.5 (FDR corrected p < 5.99× 10^−8^) even when neither marker alone exhibits noticeable survival effect (FDR corrected p < 0.01). In order to assess the relative performance of the Geninter and logrank statistic, we calculated the false positive and true positive rates for both methods when the number of affected marker pairs was varied. The false positive rate is the number of false positives divided by the sum of false positives and true negatives. The true positive rate is the number of true positives divided by the sum of true positives and false negatives. Based on the true and false positives, we calculated the receiver operating characteristic (ROC) curves for both algorithms [22]. ROC curves enable a direct comparison of true and false positive rates while varying the threshold. We analyzed the behavior of the true positive and false positive rates with independent, simulated test data. For each of the rank vectors in Figure 5, we executed the analysis with both algorithms. We increased the number of affected marker pairs and recorded the changes in true and false positives. Furthermore, we repeated each simulation 20 times for each affected marker pair number, and averaged the rates over these repetitions to account for simulation variance. Both statistics were able to identify affected marker pairs correctly. However, the false positive rate of both methods increase along with the number of affected markers (Figure 5). Furthermore, the logrank statistic has a substantially worse false positive rate indicating that most of its findings are false positives even at very low p-value thresholds. The sharp, smooth form of the logrank ROC curves in Figure 5 reflects the rise of the false positive rate of the logrank test even at p-value thresholds near zero. The p-value threshold of significance for Geninter decreases when the proportion of affected to non-affected markers increases. For a low ratio (less than10%) of affected marker pairs to non-affected marker pairs, less than 10% false positive rate and over 99% true positive rate are achieved with the nominal p-value < 0.01.

## Conclusions

We have designed and implemented a novel algorithm, Geninter, to identify SNP-pairs with combinatorial survival effect. Our results with simulated data, which is based on SNP data from 1,000 breast cancer patients, demonstrate Geninter to be both accurate and reliable. Geninter outperforms the logrank test, which is a widely used test for uncovering significant differences in survival data. Additionally, simulations where the number of samples was varied, indicate that Geninter results in a good balance of true and false positives with1,000 samples, and it is applicable to cohorts with more than 500 samples (data not shown). Given the current large-scale cancer data collection efforts, such as The Cancer Genome Atlas [23], many cancer types with thousands of samples with SNP and clinical data will be soon available and Geninter can be directly applied to such data sets.

In order to be able to analyze the billions of putative SNP combinations in the large-scale data sets, we have developed two implementations of Geninter. The R implementation allows testing and running a relatively small number of SNPs. For instance, a run with 140 markers and 1,000 samples takes approximately five hours with a standard laptop. As there are 10^10 ^SNP-SNP combinations to be computed for approximately 550k SNPs, we provide a Fortran implementation with the Message Passing Interface (MPI) that can be run on a high-performance computer cluster. We have tested the Fortran implementation of Geninter on a high-performance computer cluster at CSC-IT Center for Science. Our analysis indicates that a genome-wide analysis of all pairwise combinations of 550k markers takes approximately 1,500 hours with 256 computing nodes. The astronomical number of tests emerging from a genome-wide pair-wise analysis basically renders the FDR correction unpractical and useless. Thus, p-values are used only to sort the Geninter computed ranks. We note that Geninter is not restricted to pair-wise analysis but is applicable to any number of combinations. Obviously, higher order combinations require prior selection of attributes or other pre-processing methods to reduce the search space.

The Geninter algorithm is particularly useful in situations where the number of groups or population size is high. We have demonstrated that Geninter is able to integrate SNP-pairs to survival data. The approach, however, is applicable to other markers, such as methylation markers and copy number variants, as well. The major limiting factors for the use of Geninter are the availability of data and computational power. Given a number of large-scale efforts to quantify genetic profiles and other markers for thousands of cancer patients and exponential increase in computing power, we believe that Geninter will be a useful tool to identify combinatorial survival effects of multiple attributes, which provide a solid basis for advanced analysis of complex disorders.

## Competing interests

The authors declare that they have no competing interests.

## Authors' contributions

RL and ML designed the algorithm with TH and SL. RL implemented the algorithm with ML and PM. RL and VR wrote the formal description. MM analyzed the rank distribution. RL, ML and SH designed the simulation and interpreted results. HN, CB and JL provided materials for the simulation. HN and SH supervised the study. RL, SH, and HN wrote the manuscript. All authors participated in the critical revision of the manuscript and approved the final manuscript.

## Declarations

The publication costs for this article were funded by the Academy of Finland Center of Excellence in Cancer Genetics Research Grant Agreement n. 250345.

This article has been published as part of BMC Systems Biology Volume 7 Supplement 1, 2013: Selected articles from the 10th International Workshop on Computational Systems Biology (WCSB) 2013: Systems Biology. The full contents of the supplement are available online at http://www.biomedcentral.com/bmcsystbiol/supplements/7/S1.
